# Clinical phenotypes and genetic features of hereditary transthyretin amyloidosis patients in China

**DOI:** 10.1186/s13023-022-02481-9

**Published:** 2022-09-02

**Authors:** Xinyue He, Zhuang Tian, Hongzhi Guan, Shuyang Zhang

**Affiliations:** 1 Department of Cardiology, Peking Union Medical College Hospital, Chinese Academy of Medical Sciences and Peking Union Medical College, Beijing, People’s Republic of China; 2 Department of International Medical Service, Peking Union Medical College Hospital, Chinese Academy of Medical Sciences and Peking Union Medical College, Beijing, People’s Republic of China; 3 Department of Neurology, Peking Union Medical College Hospital, Chinese Academy of Medical Sciences and Peking Union Medical College, Beijing, People’s Republic of China

**Keywords:** Transthyretin amyloidosis, Rare disease, Hereditary, Chinese

## Abstract

**Background:**

Hereditary transthyretin amyloidosis (hATTR) is a progressive and fatal disease with heterogenous clinical presentations, limited diagnosis and poor prognosis. This retrospective analysis study aimed to report the genotypes and phenotypes of herediary transthyretin amyloidosis (hATTR) in Chinese through a systematic review of published literature.

**Methods:**

The systematic review included structured searches of peer-reviewed literature published from 2007 to 2020 of following online reference databases: PubMed, Web of Science and the literature database in China. Extracted data included sample size, personal information (sex, age, natural course, family history), mutation type, clinical milestones and reason of death.

**Results:**

We described 126 Chinese patients with hereditary transthyretin amyloidosis identified through a systematic review of 30 studies. The most common genotype in the Chinese population was Gly83Arg (25, 19.8%), which most likely presented visual and neurological abnormalities without reported death. The second and third most common genotypes were Val30Met (20, 15.9%) and Val30Ala (10, 7.9%). Peripheral neurological manifestations (91, 72%) were dominant in 126 patients. The followed manifestation was autonomic neurological abnormalities (73, 58%). Half of the cases were reported to have visual disorders, and nearly one-third of the cases presented cardiac abnormalities. Among all 126 reported patients, 46.03% were classified as neurological type, 30.16% as mixed type and only 2.38% as cardiac type. In addition. Chinese patients were mostly early onset, with age of onset at 41.8 (SD: 8.9) years, and the median time from onset to death was 7.5 [IQR: 5.3] years. Patients with cardiac involvement had a shorter survival duration (log Rank (Mantel-Cox), χ^2^ = 26.885, *P* < 0.001).

**Conclusions:**

This study focused on 126 Chinese hATTR patients obtained from a literature review. A total of 26 kinds of TTR mutations were found and the most common one was Gly83Arg. As for phenotype, 46.03% were classified as neurological type, 30.16% as mixed type and only 2.38% as cardiac type. Chinese hATTR patients were mostly early onset (AO 41.8 years), and the median time from onset to death was 7.5 years.

**Supplementary Information:**

The online version contains supplementary material available at 10.1186/s13023-022-02481-9.

## Background

Hereditary transthyretin amyloidosis (hATTR) is a rare autosomal-dominant disorder. It is characterized by the extracellular deposition of amyloid fibrils in tissues and organs such as the heart, peripheral neuropathy and eyes. Amyloidosis can be life-threatening with a short duration between onset and death [1, 2]. hATTR has been reported in 36 countries globally [3], with a prevalence of 10,186 persons (range, 5526–38,468) [4], and is especially endemic in Portugal, Sweden and Japan [5]. The prevalence of hATTR in China was estimated to be 2000 (range 435–10,134) [4].

Normal transthyretin (TTR) is a tetramer composed of four identical subunits. TTR gene mutations could cause abnormal depolymerization, misfolding and aggregation of TTR, leading to amyloidosis formation. Misfolded TTR monomers deposit in the form of amyloid fibrils and lead to intractable peripheral sensory and motor neuropathy, autonomic neuropathy, cardiomyopathy and other presentations.

More than 140 TTR mutations have been reported worldwide, and there are genotype–phenotype variations among different ethnic groups and regions. The Val30Met mutation was the most frequent mutations in European patients and was commonly associated with peripheral neuropathy [6], but those carrying the Val122Ile mutation popular in United States commonly manifest with late-onset cardiomyopathy [7]. To date, there have been few systematic reports on hATTR patients in China, especially in mainland China, although single cases or pedigrees of hATTR have been reported. There may be a distinct spectrum of TTR mutations in China since some mutations such as Ala97Ser have never been reported in Caucasian populations. And we also want to know if the predominant mutation type differs between the Chinese and the other population in East Asia. Hence, it is important to have a comprehensive and systemic review of the genotypic and phenotypic spectrum of hATTR in Chinese descent and it could help improve better understanding of this disease and might improve early screening and diagnosis in Chinese population.

In this study, we searched all related literatures on hATTR in the Chinese population and retrospectively analyzed clinical and genetic features to develop a more comprehensive view of the genotype and phenotype of Chinese hATTR patients.

## Methods

This systematic review was conducted according to the PRISMA statement for reporting systematic reviews [7].

### Search strategy

The systematic review included structured searches of peer-reviewed literature published from 2007 to 2020 via the following online reference databases: PubMed, Web of Science and the literature database in China. The searched key words included “transthyretin”, “amyloidosis”, “hereditary transthyretin amyloidosis”, “familial amyloidotic polyneuropathy (FAP)”, “familial amyloidotic cardiomyopathy (FAC) and “ATTR cardiomyopathy”. All the above key words were combined with “China or Chinese”. These searches were conducted without regard to language or geography.

### Case eligibility

Titles and abstracts were independently screened against inclusion criteria by two reviewers (HXY, TZ). Full texts of screened papers were assessed for inclusion criteria and study quality. We pilot tested eligibility criteria and included a flow diagram of study selection and reasons for exclusion to conform to the PRIMSA statement. Reports with definite TTR mutations and detailed descriptions of clinical presentations were included in our study. Duplicated reports and those lacking specific patient data were removed according to the criteria.

### Data extraction

Two authors (HXY, TZ) independently extracted data from articles and documented findings. For each identified patient, the following variables were collected: (1) personal information (sex, age, natural course, family history); (2) age at disease onset and diagnosis; (3) reported presentations and involved organ; (4) mutation type; and (5) age and reason of death.

**Fig. 1:**
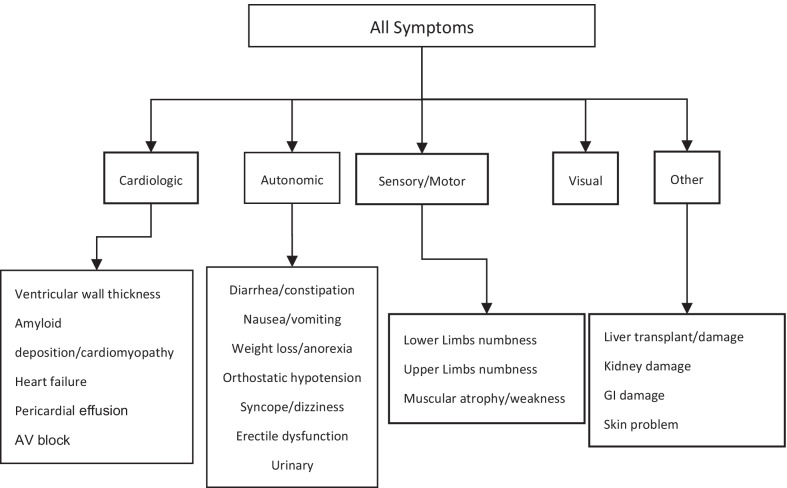
All Presentations description category

Descriptive analysis of the data included the following: (1) age at disease milestones (onset, diagnosis and death) by genotype. Subjects were classified into early- or late-onset groups depending on the onset age before or after 50 years old. (2) Reported presentations and involved organs were further categorized as peripheral neuropathy, autonomic neuropathy, cardiac or visual abnormality and others (Fig. 1). Hereditary transthyretin amyloid polyneuropathy (hATTR-PN) included peripheral neuropathy and autonomic neuropathy. The former one included paresthesia, hypesthesia, muscular atrophy and walking disability and latter one consisted of gastrointestinal dysfunction (diarrhea/constipation, nausea/vomiting and weight loss/anorexia), orthostatic hypotension, syncope or dizziness and genitourinary dysfunction (erectile dysfunction and urinary disorders). Hereditary transthyretin amyloid cardiomyopathy (hATTR-CM) had cardiac abnormalities included heart failure, conduction block, ventricular wall thickness on echocardiography and amyloid deposition on pathology. For patients contained both neuropathy involvement and cardiac involvement were categorized as hATTR-MIX. Visual abnormalities consisted of vitreous opacity, glaucoma and dry eye. Others were neuropsychiatric problems (stroke, transient ischemic attack (TIA) and mental disorders), hearing loss and leptomeningeal enhancement on magnetic resonance imaging (MRI). These clinical evaluations were used to classify subjects into one of the following three phenotypes: (1) neurological type, with peripheral or autonomic neuropathy and without cardiac involvement; (2) cardiac type, with cardiac abnormality and without or with only mild neurological abnormalities; and (3) mixed type, which covered all remaining symptomatic subjects.

### Statistical analysis

The demographic data of the cases were included for descriptive statistics. Time between disease milestones was calculated if all the values were available. Simple binary and continuous variables were shown using descriptive statistics. The normal distribution of data was tested using the Shapiro–Wilk test and expressed as mean and standard deviation (SD) or median and interquartile interval (IQR) based on the distribution. We assessed the risk of bias according to Newcastle–Ottawa Scale (NOS).

We further classified the 126 cases into two groups according to mutations and presentations: Gly83Arg mutation and non-Gly83Arg mutation; hATTR-PN and hATTR-CM and hATTR-MIX. Continuous variables were compared between two defined groups using Student’s *t*-test, while categorical variables were compared using the Chi-square test or Fisher’s exact test. Kaplan–Meier survival was calculated from the date of the disease-related presentation to the date of death.

A random effects analysis of individual studies was performed to plot the survival curve of patients with hATTR. We calculated the pooled estimate of 2, 5 and 10-year survival for each patient subgroup.

All tests were 2-tailed, *P* value of < 0.05 was considered statistically significant. Statistical analyses were performed by using RGui (version 4.2.0).

## Results

### Study characteristics

**Fig. 2:**
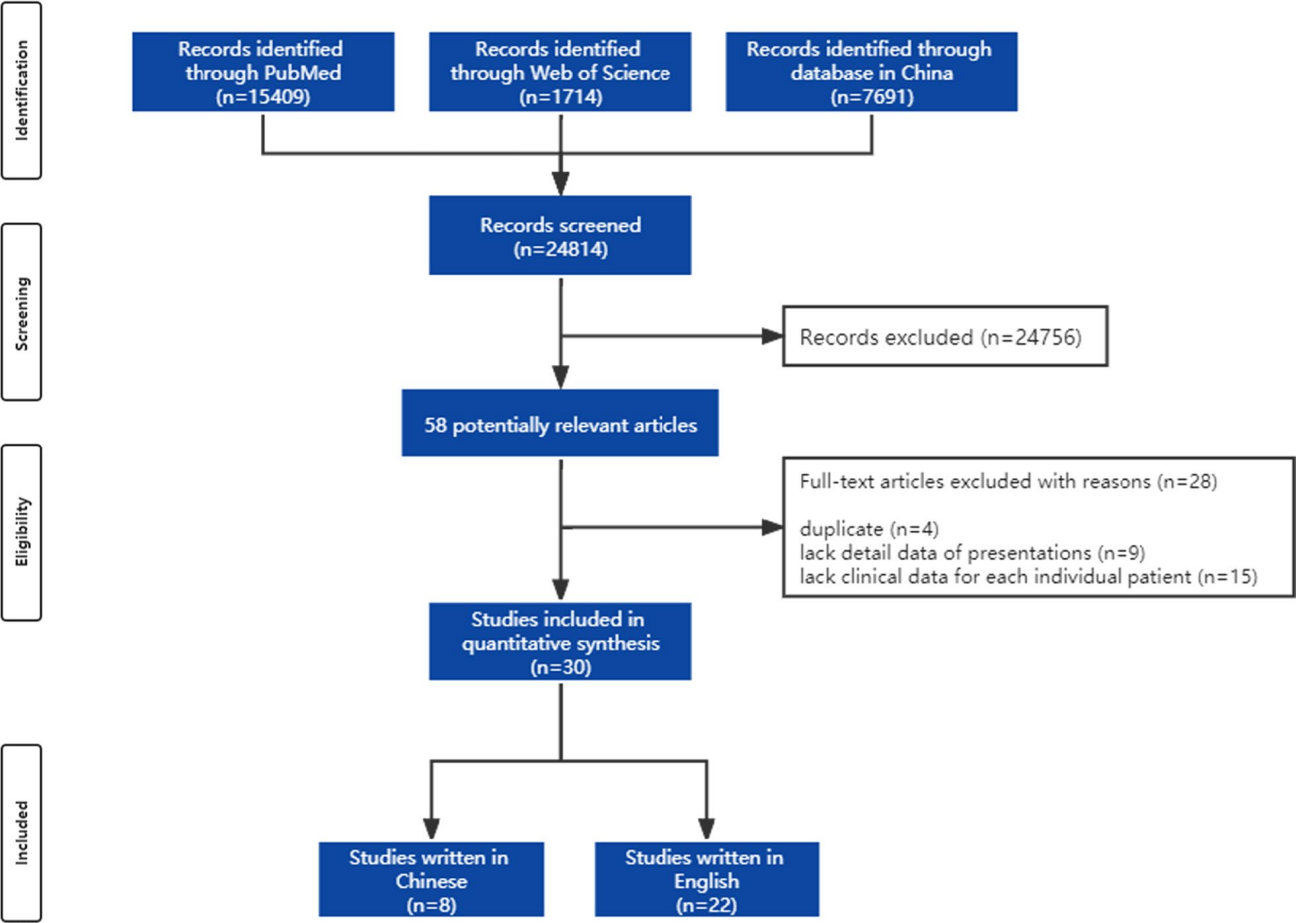
Case eligibility

The initial search yielded 24,814 results: 15,409 on PubMed, 1714 on Web of Science and 7691 on literature databases in China. Of these, 24,756 were excluded by only reading the titles and abstracts. There were 58 potentially relevant reports extracted from the databases and read fully. After applying eligibility criteria and removing duplicate reports, 28 reports were excluded. Among these reports, 4 reports were duplicates, 9 reports did not include the detailed description of presentations, and 15 reports could not discriminate the demographics and clinical data for each individual patient (Fig. 2). In the remaining 30 individual reports, 8 reports were written in Chinese, and the other 22 reports were written in English (Table 1).

**Table 1:** The baseline characteristics of the included studies

No	Author, year	Sample size	Genotype	Title	Source
1	Xu et al. [18]	7	Val30Ala	Transthyretin Val30Ala mutation in a Chinese family with familial amyloid polyneuropathy: clinical, pathological and genetic investigation	Chinese Journal of Neurology
2	Li et al. [13]	24	Val30Met	Clinical and genetic analysis of three families with familiar amyloid polyneuropathy	Chinese Medical Sciences Journal
			Phe33Val		
			Gly67Glu		
3	Xi et al. [30]	1	Glu54Lys	A Case of Familial Amyloid Polyneuropathy with a Transthyretin Glu54Lys Mutation	Chinese Journal of Clinical Neurosciences
4	Liu et al. [17]	1	Val30Ala	Clinical and histopathological features of familial amyloidotic polyneuropathy with transthyretin Val30Ala in a Chinese family	Journal of the Neurological Sciences
5	Zhang et al. [35]	1	Tyr114Cys	Transthyretin-Related Hereditary Amyloidosis in a Chinese Family with TTR Y114C Mutation	Neuro-degenerative diseases
6	Chen et al. [8]	2	Gly83Arg	Transthyretin Arg-83 mutation in vitreous amyloidosis	International Journal of Ophthalmology
7	Long et al. [21]	15	Lys35Thr	Vitreous amyloidosis in two large mainland Chinese kindreds resulting from transthyretin variant Lys35Thr and Leu55Arg	Ophthalmic genetics
			Leu55Arg		
8	Zhang et al. [12]	9	Gly83Arg	Mutation p.G83R in the transthyretin gene is associated with hereditary vitreous amyloidosis in Han Chinese families	Molecular Vision
9	Zou et al. [22]	6	Ala36Pro	Transthyretin Ala36Pro mutation in a Chinese pedigree of familial transthyretin amyloidosis with elevated vitreous and serum vascular endothelial growth factor	Experimental Eye Research
10	Xie et al. [11]	1	Gly83Arg	Family with familial vitreous amyloidosis with thyroid hormone binding protein Gly83Arg mutation	Chinese Journal of Ocular Fundus Diseases
11	Liu et al. [10]	12	Gly83Arg	Ophthalmic manifestations in a Chinese family with familial amyloid polyneuropathy due to a TTR Gly83Arg mutation	Eye
12	Yin et al. [9]	1	Gly83Arg	Chinese familial transthyretin amyloidosis with vitreous involvement is associated with the transthyretin mutation Gly83Arg: a case report and literature review	Amyloid
13	Fan et al. [23]	1	Gly47Arg	Myocardial involvement in transthyroxine protein-associated amyloidosis: a case report	Chinese Journal of Cardiology
14	Lv et al. [15]	1	Ile107Met	Multimodal retinal imaging in a Chinese kindred with familial amyloid polyneuropathy secondary to transthyretin Ile107Met mutation	Eye
15	Guan et al. [19]	13	Val30Ala	Clinical, neuropathological and genetic findings in patients with transthyretin-associated familial amyloid polyneuropathy	Chinese Journal of Neurology
			Val30Met		
			Phe33Leu		
			Lys35Asn		
			Asp38Val		
			Gly47Arg		
			Gly53Glu		
			Glu54Gln		
			Glu54Lys		
16	Meng et al. [2]	9	Val30Met	Hereditary Transthyretin Amyloidosis in Eight Chinese Families	Chinese Medical Journal
			Phe33Leu		
			Ala36Pro		
			Val30Ala		
			Phe33Val		
			Glu42Gly		
17	Chen et al. [16]	1	Val50Leu	Exome Sequencing and Gene Prioritization Correct Misdiagnosis in a Chinese Kindred with Familial Amyloid Polyneuropathy	Scientific Reports
18	Cheng et al. [16]	1	Val50Met	Late-onset familial amyloid peripheral neuropathy: a case report and literature review	Chinese Journal of Nervous and Mental Diseases
19	Hu et al. [27]	1	Ala117Ser	Familial amyloid cardiomyopathy masquerading as chronic Guillain–Barre syndrome: things are not always what they seem	Frontiers of Medicine
20	Liu et al. [32]	5	Thr49Ala	Clinical features of familial amyloid polyneuropathy carrying transthyretin mutations in four Chinese kindreds	Journal of the Peripheral Nervous System
			Leu55Arg		
			Tyr116Ser		
			Ala36Pro		
21	Xu et al. [33]	1	Leu75Pro	Transthyretin-related hereditary amyloidosis with recurrent vomiting and renal insufficiency as the initial presentation A case report	Medicine
22	Yang et al. [31]	1	Val50Leu	Clinical and electrophysiological features of familial amyloid polyneuropathy induced by TTR Val50Leu mutation	Chinese Journal of Neuroimmunology and Neurology
23	Chen et al. [34]	1	Leu75Pro	Liver transplantation for the treatment of hereditary amyloidosis of transthyroid protein: a case report	Chinese Journal of Hepatobiliary Surgery
24	Zhu et al. [14]	1	Lys35Thr	Pathogenic gene mutation in a Han Chinese family with hereditary vitreous amyloidosis identified by Sanger sequencing	Chinese Journal of Clinical Laboratory Science
25	Chen et al. [26]	1	Ala117Ser	A Missense Variant p.Ala117Ser in the Transthyretin Gene of a Han Chinese Family with Familial Amyloid Polyneuropathy	Molecular Neurobiology
26	Hu et al. [37]	1	Glu74Lys	A Family Report and Literature Review of TTR-type FAP with Eye Abnormalities	Journal of Apoplexy and Nervous Diseases
27	Yuan et al. [29]	1	Ala117Ser	Familial amyloid polyneuropathy with chronic paroxysmal dry cough in Mainland China: A Chinese family with a proven heterozygous missense mutation c.349G > T in the transthyretin gene	Journal of Clinical Neuroscience
28	Fan et al. [25]	5	Asp18Gly	The identification of a transthyretin variant p.D38G in a Chinese family with early-onset leptomeningeal amyloidosis	Journal of Neurology
29	Miao [36]	1	Phe84Ser	A case of amyloid peripheral neuropathy with central involvement caused by phe84ser mutation	Chinese Journal of Integrative Medicine on Cardiocerebrovascular Disease
30	Qin [28]	1	Ala117Ser	Noninvasive diagnosis of hereditary transthyretin-related cardiac amyloidosis: A case report	Medicine

### Demographic data

The retained reports included 126 cases contributed by 58 pedigrees of Han Chinese descent. A formal bias assessment of identified studies was provided in Additional file 1: Table S1. Genotypes were accurately identified in the above reports. Among the 126 cases, the description analysis was conducted by different genotypes, including the age of onset, death and duration between onset to diagnosis or death (Table 2). Of all 126 cases, 87 (69.0%) patients were male, and 104 (82.5%) had a family history. The mean age at onset (AO) and disease duration from presentation to diagnosis were 41.8 ± 8.9 years and 5.9 ± 3.6 years, respectively. The median duration from presentation to death was 7.5 years, with a mean age of death (AD) of 47.2 years. Leu55Arg showed a clearly better prognosis, with a duration from presentation to death of 27.0 [IQR: 16.0] years.

**Table 2:** Characteristics of 126 hATTR cases

	All	Gly83Arg	Val30Met	Val30Ala	Leu55Arg	Ala36Pro	Lys35Thr	Gly47Arg	Asp18Gly	Gly47Glu	Ala117Ser	Other^a^
		(p.Gly103Arg)	(p.Val50Met)	(p.Val50Ala)	(p.Leu75Arg)	(p.Ala56Pro)	(p.Lys55Thr)	(p.Gly67Arg)	(p.Asp38Gly)	(p.Gly67Glu)	(p.Ala137Ser)	
N	126	25	20	10	9	9	8	6	6	6	4	23
% of samples	100.0%	19.8%	15.9%	7.9%	7.1%	7.1%	6.3%	4.8%	4.8%	4.8%	3.2%	18.3%
% Male	87 (69.0%)	13 (52.0%)	15 (75.0%)	4 (40.0%)	7 (77.8%)	7 (77.8%)	5 (62.5%)	5 (83.3%)	4 (66.7%)	6 (100.0%)	3 (75.0%)	18 (78.3%)
% Family history	104 (82.5%)	25 (100.0%)	11 (55.0%)	10 (100.0%)	9 (100.0%)	6 (66.7%)	8 (100.0%)	5 (83.3%)	6 (100.0%)	6 (100.0%)	2 (50.0%)	16 (69.6%)
Age at presentation onset, years	41.8 (SD: 8.9)	39.4 (SD: 4.1)	47.0 (SD: 8.6)	33.0 [IQR: 9.5]	33.9 (SD: 5.4)	40.0 [IQR: 19.0]	44.3 (SD: 2.9)	29.5 [IQR: 8.0]	31.0 [IQR: 14.5]	40.0 (SD: 6)	60.8 (SD: 4.8)	40.3 (SD: 8.2)
Late -onset, n (%)	23 (18.3%)	9 (36.0%)	6 (30.0%)	0 (0)	0 (0)	0 (0)	0 (0)	0 (0)	0 (0)	0 (0)	4 (100.0%)	4 (17.4%)
Time from presentation onset to diagnosis, years	5.9 (SD: 3.6)	8.5 (SD: 5.3)	8.6 (SD: 2.3)	2.0 [IQR: 4.0]	6.3 (SD: 2.3)	2 (SD: 0.9)	2.4 (SD: 0.6)	3 (SD: 0.8)	4.5 (SD: 0.5)	3.5 (SD: 0.5)	3.5 (SD: 1.5)	4.6 (SD: 2.9)
Age at Death, years	47.2 (SD: 10.2)		54.3 (SD: 3.8)	37.0 [IQR: 5.5]	58.0 [IQR: 15.0]	58.7 (SD: 1.8)	57.7 (SD: 3.1)	33.0 [IQR: 4.0]	39 (SD: 4.0)	45.5 (SD: 6.5)	–	39 [IQR: 4.0]
Time from presentation Onset to death, years	7.5 [IQR: 5.3]	–	8.7 (SD: 0.6)	4.0 [IQR: 4.0]	27.0 [IQR: 16.0]	10 (SD: 1.3)	12.7 (SD: 4.0)	4.7 [IQR: 1.5]	6.0 [IQR: 13.5]	4.5 [IQR: 1.0]	–	4.5 [IQR: 1.0]

^a^ Genotypes with no more than 3 subjects.including Phe33Val (three subjects); Glu54Lys, Phe33Leu, Val50Leu, Thr49Ala, Leu75Pro (two subjects each), Tyr114Cys, Ile107Met, Lys35Asn, Asp38Val, Gly53Glu, Glu54Gln, Glu42Gly, Tyr116Ser, Phe84Ser, Glu74Lys (one subject each); For descriptive analysis, mean and standard deviation (SD) were calculated for normal distribution, while median and inter-quartile range [IQR] were calculated for abnormal distribution

### Genetic results

Genotype analysis revealed 26 TTR mutations, including Gly83Arg [8–12] (25 cases), Val30Met [13–16] (20), Val30Ala [17–19](10), Leu55Arg [20, 21](9), Ala36Pro [22](9), Lys35Thr [21](8), Gly47Arg [19, 23](6) Asp18Gly [24, 25](6), Gly47Glu [13] (6), Ala117Ser [26–29](4) and other mutations in less than 4 subjects each, including Phe33Val [2] (three subjects); Glu54Lys [19, 30], Phe33Leu [15, 18], Val50Leu [16, 31], Thr49Ala [32], Leu75Pro [33, 34] (two subjects each) and Lys35Asn [19], Asp38Val [14],Glu42Gly [2], Gly53Glu [14], Glu54Gln [14], Ile107Met [15], Tyr114Cys [35], Tyr116Ser [32], Phe84Ser [36], Glu74Lys [37] (one subject each). The three most common genotypes were Gly83Arg (19.8%), Val30Met (15.9%) and Val30Ala (7.9%).

### Clinical milestones and survival analysis

A summary of meaningful milestones was listed in Table 2. The mean AO was 41.8 years. The Ala117Ser cases were all late-onset, and the mean AO was 60.8 ± 4.8 years. The average AO of other mutations was less than 50 years old. Nine cases (36%) of Gly83Arg mutation, six (30.0%) of Val30Met, one of Phe33Leu (50%) and Lys35Asn, Ile107Met and Tyr116Ser mutation (100%) were late-onset, while other mutations (Asp18Gly, Val30Ala, Phe33Val, Lys35Thr, Ala36Pro, Asp38Val, Glu42Gly, Gly47Arg,, Gly47Glu, Thr49Ala, Val50Leu, Gly53Glu, Glu54Gln, Glu54Lys, Leu55Arg, Glu74Lys, Leu75Pro, Phe84Ser and Tyr114Cys) were only early-onset.

**Fig. 3:**
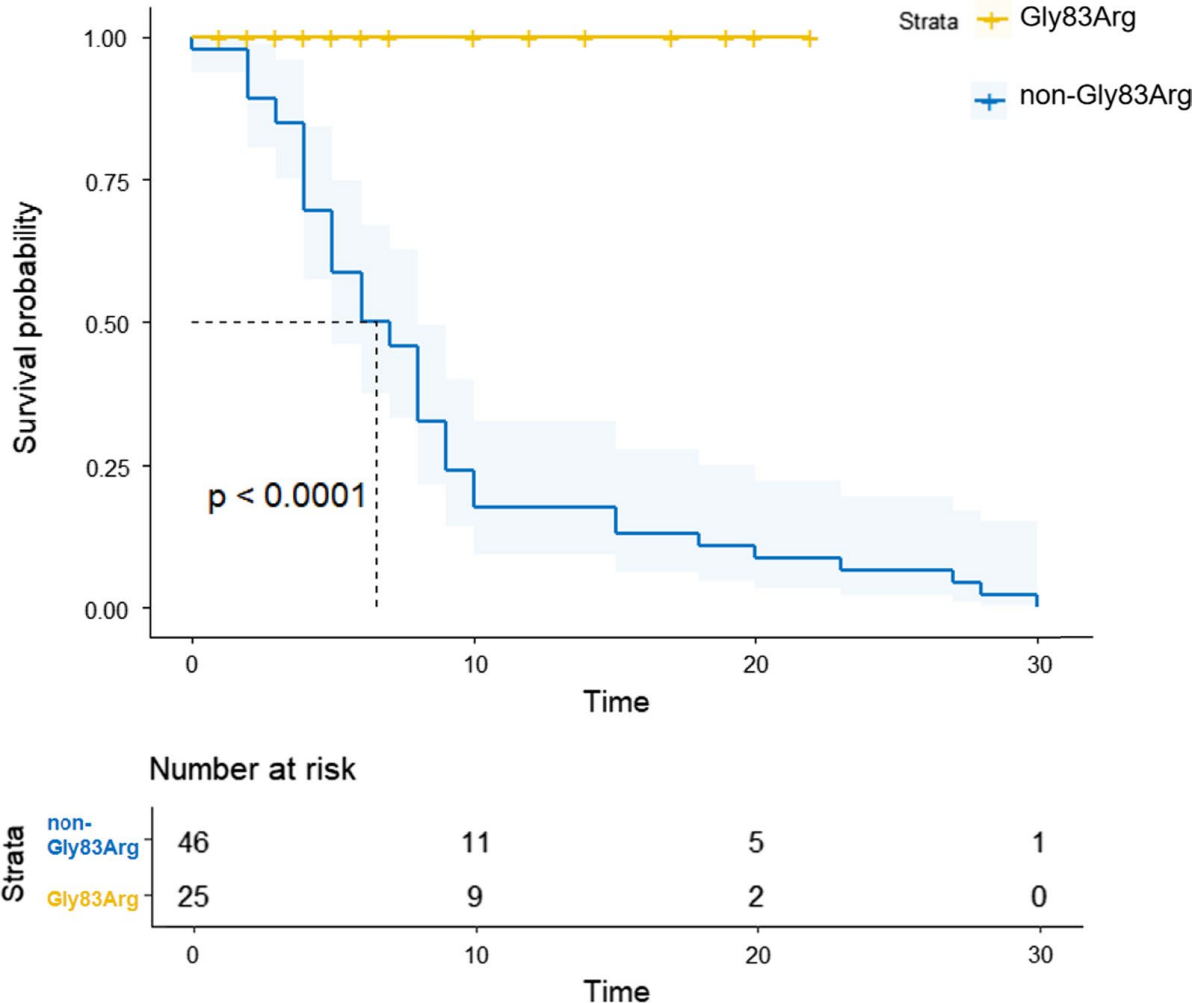
Kaplan–Meier survival curves of data obtained from symptom onset. We have divided the hATTR patients into two groups: Gly83Arg and non-Gly83Arg. The total mortality of non-Gly83Arg was 45.5% and the median survival time from symptom onset was 7.5 (IQR: 5.3) years. The evidence showed a clear better survival in patients with Gly83Arg mutation (log Rank (Mantel-Cox), χ^2^ = 24.383, *P* < 0.001)

Patients with hATTR had a poor prognosis, and the mean age of death was 47.2 years. We further divided the patients into two groups: Gly83Arg and non-Gly83Arg. A Kaplan–Meier analysis was performed, as shown in Fig. 3. And analysis for mean age of onset and mean age of diagnosis of Gly83Arg mutations in transthyretin amyloidosis of different studies was added in Additional file 1: Fig. S1. The evidence showed a clear better survival in patients with Gly83Arg mutation (log Rank (Mantel-Cox), χ^2^ = 24.383, *P* < 0.001). Patients with the Gly47Arg mutation had the lowest AD (33.0 [IQR: 4.0] years), and those with the Ala36Pro mutation had the maximum AD (58.7 ± 1.8 years). No deaths were reported in patients with Gly83Arg and Ala117Ser mutations. Patients with the Leu55Arg mutation had the longest duration from onset to death (27.0 [IQR: 16.0] years). Patients with Lys35Thr and Ala36Pro had longer survival times (12.7 ± 4.0 and 10 ± 1.3 years, respectively), while Val30Ala, Gly47Glu and Gly47Arg had worse prognosis (survival times were 4.0, 4.5 and 4.7 years, respectively). Patients with the Val30Ala mutation had the shortest time from presentation onset to death (4.0 [IQR: 4.0] years). Gly47Arg mutation had the lowest AO (29.5 [IQR: 8.0] years) and AD (33.0 [IQR: 4.0] years) and a shorter duration from onset to death (4.7 [IQR: 1.5] years).

**Fig. 4:**
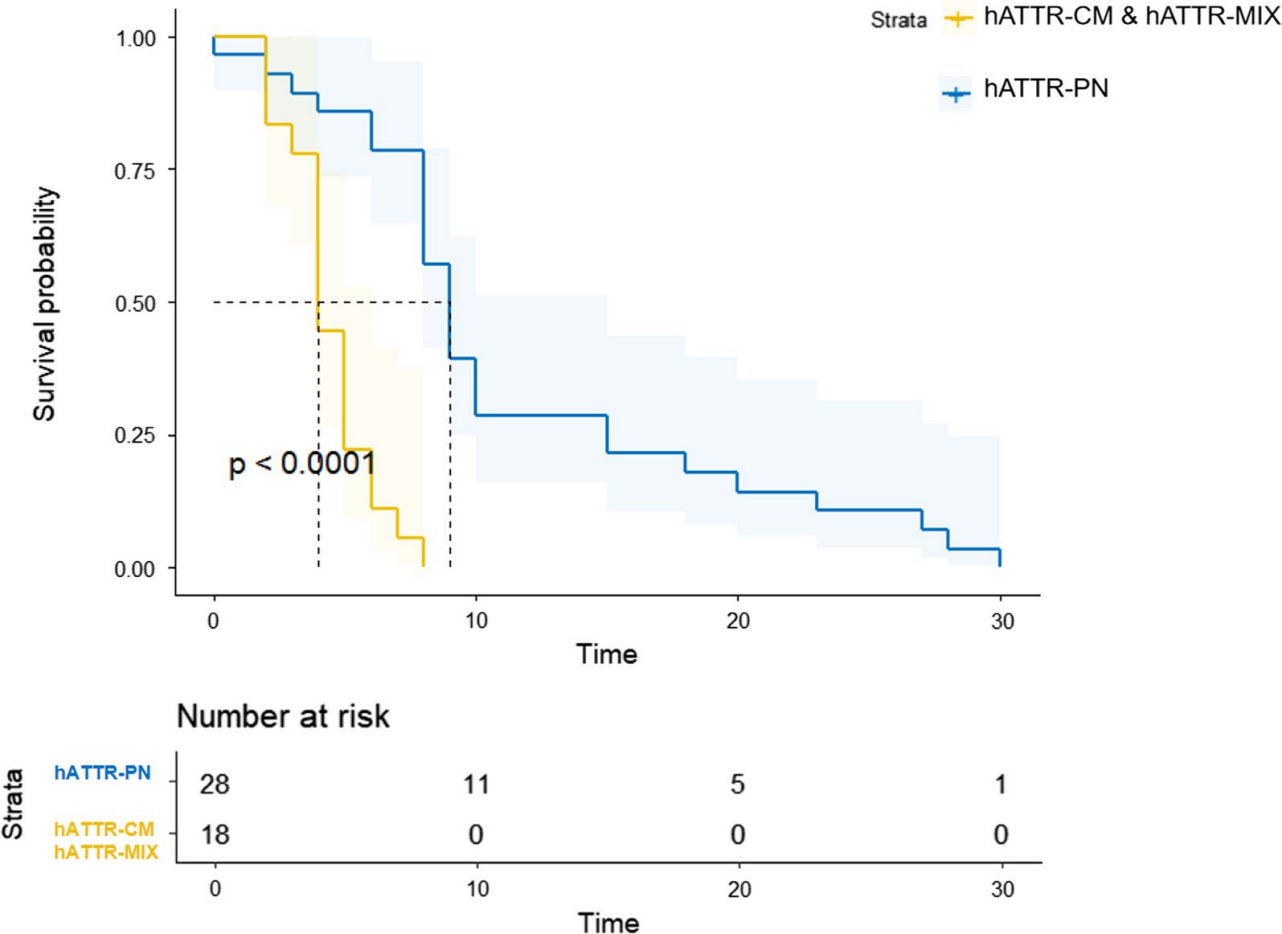
Kaplan–Meier survival curves of data obtained from symptom onset. We have divided the hATTR patients into two groups: Cardiac-involve (hATTR-CM and hATTR-MIX) and Non-cardiac-involve (hATTR-PN). The evidence showed a clear worse survival in patients with cardiac-involvements (log Rank (Mantel-Cox), χ^2^ = 26.885, *P* < 0.001)

Patients had cardiac involvement showed a clear worse prognosis than patients with only neuropathy presentations. A Kaplan–Meier analysis was performed, as shown in Fig. 4 (log Rank (Mantel-Cox), χ^2^ = 26.885, *P* < 0.001). And the estimate of heterogeneity was taken as random-effect model. The estimated survival of hATTR patients was 94.0% (95% CI 87.5–100.0) at 2-year, 59.5% (95% CI 35.8–83.2) at 5-years and 28.5% (95% CI 6.8–50.3) at 10-years. The original forest plots were shown in the Additional file 1: Fig. S2.

### Association of clinical presentations and genotype

The phenotypes by different genotypes were listed in Table 3. Neurological phenotype was the most common one in the 126 patients. Ninety-one cases (72.22%) presented peripheral neurological problems, which were higher in the Val30Ala (100%), Gly47Arg (100%), Ala117Ser (100%), Val30Met (100%), Gly47Glu (83.3%) and Ala36Pro (77.8%) mutations than in the Leu55Arg (44.4%), Gly83Arg (40%) and Asp18Gly (16.7%) mutations. Autonomic neurological presentations (57.9%) were the second most common. Val30Ala (100%), Gly47Arg (100%), Gly47Glu (83.3%), Val30Met (100%) and Ala117Ser (75%) had obviously higher rates of these autonomic abnormalities than the Gly83Arg (0), Lys35Thr (0) and Asp18Gly (0) mutations. Thirty-nine cases (31%) presented cardiac abnormalities. All patients with Val30Ala and Gly47Arg mutations had cardiac problems, while those with Leu55Arg (0%), Lys35Thr (0%), Asp18Gly (0%) and Gly83Arg (4%) had no or very few cardiac abnormalities. Sixty-three cases (50%) were reported to have visual disorders. Gly83Arg (100%) and Leu55Arg (100%) contributed most of the visual presentations, followed by Lys35Thr (87.5%), Ala36Pro (77.8%) and Gly47Arg (50%). Asp18Gly (0), Gly47Glu (0), Ala117Ser (0), Val30Met (5.0%) and Val30Ala (10%) had no or very few visual problems. Eighteen cases were reported with other presentations, followed by leptomeningeal enhancement (5.6%), neuropsychiatric problems (4.8%) and hearing loss (4.0%). Asp18Gly mutant was liable to have neuropsychiatric problems (83.3%) and leptomeningeal enhancement (33.3%), and Gly47Arg also had leptomeningeal enhancement (33.3%).

**Table 3:** Presentation distributions of 126 hATTR cases

Feature	All	Gly83Arg	Val30Met	Val30Ala	Leu55Arg	Ala36Pro	Lys35Thr	Gly47Arg	Asp18Gly	Gly47Glu	Ala117Ser	Other^a^
		(p.Gly103Arg)	(p.Val50Met)	(p.Val50Ala)	(p.Leu75Arg)	(p.Ala56Pro)	(p.Lys55Thr)	(p.Gly67Arg)	(p.Asp38Gly)	(p.Gly67Glu)	(p.Ala137Ser)	
N	126	25	20	10	9	9	8	6	6	6	4	23
Cardiologic	39 (30.95%)	1	3	10	0	2	0	6	0	4	2	11
Autonomic	73 (57.94%)	0	20	10	4	4	0	6	0	5	3	21
Sensory/Motor	91 (72.22%)	10	20	10	4	7	6	6	1	5	4	18
Visual	63 (50.00%)	25	1	1	9	7	7	3	0	0	0	10
Other	18 (14.29%)	0	0	4	2	0	0	2	5	0	0	5
Skin problem	3 (2.38%)	0	0	1	0	0	0	0	0	0	0	2
Neuropsychiatric problem	6 (4.76%)	0	0	0	0	0	0	0	5	0	0	1
Hearing loss	5 (3.97%)	0	0	2	2	0	0	0	1	0	0	0
Leptormeningeal enhancement	7 (5.56%)	0	0	1	0	0	0	2	2	0	0	2
*Main*												
Neurological	58 (46.03%)	9	17	0	4	4	6	0	6	0	2	10
Cardiac	3 (2.38%)	0	1	0	0	0	0	0	0	0	0	2
Mixed	38 (30.16%)	1	2	10	0	2	0	6	0	4	2	11

^a^ Genotypes with no more than 3 subjects.including Phe33Val (three subjects); Glu54Lys, Phe33Leu, Val50Leu, Thr49Ala, Leu75Pro (two subjects each), Tyr114Cys, Ile107Met, Lys35Asn, Asp38Val, Gly53Glu, Glu54Gln, Glu42Gly, Tyr116Ser, Phe84Ser, Glu74Lys (one subject each)

Among all 126 reported patients, 46.03% were classified as neurological type, 30.16% as mixed type and only 2.38% as cardiac type. One Val30Met (5%) and two Leu75Pro (100%) mutants were cardiac type. Gly83Arg mutant had mainly visual problems, partly (40%) combined with peripheral neurology, and only one (4%) had cardiac hypertrophy and abnormal late enhancement on magnetic resonance imaging. Leu55Arg and Lys35Thr mutants had neurological and visual abnormalities without any cardiac involvement. Asp18Gly mutant had mainly neuropsychiatric problems without cardiac involvement. Val30Met and Ala36Pro mutants had mainly neurological problems with a few cardiac abnormalities. Val30Ala, Gly47Arg and Gly47Glu mutants had nearly equal neurological and cardiac problems (Tables 4 and 5).

**Table 4:** Neurological presentation distributions of hATTR cases

Feature	All	Gly83Arg	Val30Met	Val30Ala	Leu55Arg	Ala36Pro	Lys35Thr	Gly47Arg	Asp18Gly	Gly47Glu	Ala117Ser	Other^a^
		(p.Gly103Arg)	(p.Val50Met)	(p.Val50Ala)	(p.Leu75Arg)	(p.Ala56Pro)	(p.Lys55Thr)	(p.Gly67Arg)	(p.Asp38Gly)	(p.Gly67Glu)	(p.Ala137Ser)	
N	126	25	20	10	9	9	8	6	6	6	4	23
Sensory/motor	91	10	20	10	4	7	6	6	1	5	4	18
Lower limbs numbness	80	4	20	6	4	6	6	6	1	5	4	18
Upper limbs numbness	80	10	20	2	4	7	5	6	0	5	3	18
Muscular atrophy/weakness	69	0	20	4	4	4	6	6	0	5	4	16
Autonomic	73	0	20	10	4	4	0	6	0	5	3	21
Diarrhea/constipation	52	0	19	8	4	3	0	2	0	0	2	14
Nausea/vomiting	29	0	16	8	0	0	0	0	0	0	0	5
Weight loss/anorexia	26	0	2	8	0	0	0	2	0	0	1	13
Orthostatic hypotension	41	0	4	9	0	3	0	4	0	4	1	16
Syncope/dizziness	15	0	0	2	0	0	0	4	0	4	1	4
Erectile dysfunction	21	0	12	0	0	0	0	0	0	4	0	5
Urinary	25	0	16	1	0	0	0	0	0	0	0	8

^a^ Genotypes with no more than 3 subjects.including Phe33Val (three subjects); Glu54Lys, Phe33Leu, Val50Leu, Thr49Ala, Leu75Pro (two subjects each), Tyr114Cys, Ile107Met, Lys35Asn, Asp38Val, Gly53Glu, Glu54Gln, Glu42Gly, Tyr116Ser, Phe84Ser, Glu74Lys (one subject each)

**Table 5:** Cardiologic presentation distributions of hATTR cases

Feature	All	Gly83Arg	Val30Met	Val30Ala	Leu55Arg	Ala36Pro	Lys35Thr	Gly47Arg	Asp18Gly	Gly47Glu	Ala117Ser	Other^a^
		(p.Gly103Arg)	(p.Val50Met)	(p.Val50Ala)	(p.Leu75Arg)	(p.Ala56Pro)	(p.Lys55Thr)	(p.Gly67Arg)	(p.Asp38Gly)	(p.Gly67Glu)	(p.Ala137Ser)	
N	126	25	20	10	9	9	8	6	6	6	4	23
Cardiologic	39	1	3	10	0	2	0	6	0	4	2	11
Ventricular wall thickness	36	1	2	9	0	2	0	6	0	4	2	10
AV block/bundle-branch block	10	1	1	6	0	0	0	0	0	0	1	1
Heart failure	9	0	0	0	0	1	0	0	0	4	2	2
Pericardial effusion	1	0	0	0	0	0	0	0	0	0	0	1
Amyloid deposition	1	0	0	1	0	0	0	0	0	0	0	0

^a^ Genotypes with no more than 3 subjects.including Phe33Val (three subjects); Glu54Lys, Phe33Leu, Val50Leu, Thr49Ala, Leu75Pro (two subjects each), Tyr114Cys, Ile107Met, Lys35Asn, Asp38Val, Gly53Glu, Glu54Gln, Glu42Gly, Tyr116Ser, Phe84Ser, Glu74Lys (one subject each)

### Gly83Arg, the most common mutation in the Chinese population

According to the data, Gly83Arg was the most common genotype in the Chinese hATTR. Although the AO and AD in the Gly83Arg and non-Gly83Arg genotype showed no statistical significance, there was a significant difference in clinical features between Gly83Arg and the other genotype in Table 6. Analysis for Gly83Arg mutations in transthyretin amyloidosis of different studies was added in Additional file 1: Fig. S1. All patients with the Gly83Arg mutation had a family history, while only 78.22% of patients with other genotype had a family history. Patients with Gly83Arg had more visual presentations and fewer autonomic, peripheral neurological and cardiac presentations. No death was reported in Gly83Arg. The mean age of death in the non-Gly83Arg group was 47.2 years, and the median year of onset to death was 7.5 years (Fig. 3).

**Table 6:** The comparison of disease milestones and clinical presentations between Gly83Arg and non-Gly83Arg

Feature	Gly83Arg (p.Gly103Arg)	Other genotypes	*P* value
N	25	101	
% Male	52.00%	73.27%	0.07
% Family history	100.00%	78.22%	< 0.01
Age at presentation onset, years, mean (SD)	39.36 (4.06)	42.39 (13.01)	0.69
Age at diagnosis, years, mean (SD)	47.76 (7.88)	46.81 (15.12)	0.49
Age at death, years, mean (SD)	–	47.19 (10.23)	–
Time from presentation onset to diagnosis, years, mean (SD)	8.50 (5.30)	5.32 (5.90)	< 0.01
Time from presentation onset to death, years, median (IQR)	–	7.50 (5.30)	–
Autonomic	0 (0.00%)	73 (72.28%)	< 0.01
Sensory/motor	10 (40.00%)	81 (80.20%)	< 0.01
Cardiologic	1 (4.00%)	38 (37.62%)	< 0.01
Visual	25 (100.00%)	38 (37.62%)	< 0.01
Other	0 (0.00%)	18 (17.82%)	0.02

## Discussion

More than 140 TTR mutations have been reported worldwide, with varied clinical presentations and disease progression. The genotypes and phenotypes of 126 Chinese patients with hATTR were analyzed in the study in order to better understand the clinical and genetic characteristics of these patients. The three most common genotypes were Gly83Arg (19.8%), Val30Met (15.9%) and Val30Ala (7.9%). Most patients (81.7%) were early-onset. Peripheral neuropathy was the most common (72.2%) clinical presentation. The Chinese hATTR patients had the same poor prognosis, with a mean age of death of less than 50 years.

In this systematic review, we found several common and unique clinical features of Chinese hATTR patients compared to those in Europe and the United States. Apart from male predominance similar to previous studies, the DNA mutation spectrum, milestones and clinical presentations were different from previous epidemiological statistics. Gly83Arg was the most common mutation in Chinese hATTR. All mutations showed a younger mean age of disease onset than that in Europe. The delay in the diagnosis of hATTR was common in China.

Similar to previous studies [6, 38], we discovered a male predominance and not 100% family history. The TTR gene is located on chromosome 18q12.1, and hATTR is an autosomal dominant inherited disease. However, a male predominance was reported worldwide up to 2:1, instead of 1:1, as predicted. According to Urban Hellman’s study in 2008 [39], the penetrance was significantly higher with mutation genes from the mother than from the father. The gender predominance and the lack of ensured family history may be explained by the variety of mutation penetration. Among the 26 mutations identified in our study, Gly83Arg (19.8%) was the most common gene mutation and Val30Met (15.9%) was the second most common one, while this mutation was the most common in many other countries [6]. Two widely reported mutations (Ile68Leu and Val122Ile) were not found in our study. This finding suggests that geography and race can cause heterogeneity in genotypes. Other common gene mutations in the Chinese hATTR pateints include Asp18Gly, Ala36Pro, and Gly47Arg. Four (3.2%) patients with mutations of Ala117Ser were found in our review. This mutation was rarely seen and reported worldwide [40] and never in Caucasian populations. Ala117Ser had a better prognosis with no death report.

The main symptom of Gly83Arg, the most common genotype in China, was visual abnormality with some sensory/motor neurological symptoms, and few cardiac involvements. No death was reported for this genotype. There have been a few reports on Gly83Arg outside China, making it difficult to do the comparison. The better prognosis of Gly83Arg might associated with less cardiac involvement and neuropsychiatric problems.

Sensory and motor neurological presentations were the most common abnormalities in China and other countries. In our study, the neurological phenotype was observed mainly in patients with Val30Ala, Gly47Arg, Gly47Glu, Ala117Ser and Val30Met, while it is seen mainly in Val30Met mutations worldwide [41]. Val30Ala (100%), Gly47Arg (100%), Gly47Glu (66.7%) and Ala117Ser (50%) were prone to cardiac involvement. According to THAOS [6], Val30Met and Val122Ile [6] were the most common cardiac phenotypes. Regarding visual involvement, Gly83Arg (100%), Leu55Arg (100%), Lys35Thr (87.5%) and Ala36Pro (77.8%) were the most common genotypes. According to previous epidemiological studies, mutations causing visual impairment varied, and Ala36Pro was reported in Greek [42].

There were also important findings about the timing of disease milestones. The mean AO in Chinese hATTR patients was 41.8 years, whereas the onset age reported was 53 to 61 years in Western Europe [6], 70 years in the United States [38] and 51.1 years old in Japan [39]. A total of 18.3% of patients in our study were late-onset phenotype, mostly seen in the Gly83Arg (9/23), Val30Met (6/23) and Ala117Ser (4/23) genotypes. According to the THAOS registry, the late-onset phenotype was mostly seen in Val30Met, followed by Val122Ile [6], Thr60Ala and Ile68Leu in Western Europe and the United States [6]. The median time from onset to death was 7.5 years in our study, ranging from 4.0 years in the Val30Ala mutation to 27.0 years in the Leu55Arg mutation. Subjects with cardiac involvement, such as Val30Ala, Gly47Arg and Gly47Glu, had a shorter survival duration. Patients with cardiac presentations turned out to have a worse prognosis than patients with only neurological involvements. Majority of patients with hATTR-CM suffered from serious heart failure in the late course and some died of the disease of cardiac insufficiency.

The diagnosis of hATTR is not optimistic in China. The time from symptom onset to diagnosis was 5.9 years. Val30Met had the longest duration between onset and final diagnosis. The delay of proper diagnosis, even for patients with a definite family history, may be the reason for poor prognosis. Therefore, it is very important and urgent to raise awareness of hATTR in China.

This study was a patient-pool study with incomplete data, which resulted in limitations in three aspects. First, many reports were pedigrees, which would affect the distribution of genotypes. Second, the data were biased due to a lack of a comprehensive examination of disease involvement among some studies. Third, follow-up information was limited, and only some studies reported survival information.

Despite some limitations, these case reports are important resources for understanding the current situation of hATTR in China. The epidemiological characteristics of diseases vary greatly in different countries and regions. There have been few cohort studies on this rare disease in China, and our study might partially compensate for this shortcoming. However, prospective cohort studies with more samples are needed to determine the characteristics of Chinese hATTR patients.

## Conclusion

This study focused on 126 Chinese hATTR patients obtained from a literature review. The most common genotype in the Chinese population was Gly83Arg, which most likely presented with visual and neurological abnormalities without reported death. Other common genotypes were Val30Met and Val30Ala. Regarding phenotype, 46.03% were classified as neurological type, 30.16% as mixed type and only 2.38% as cardiac type. Peripheral neurological manifestations (72%) were the dominant phenotype in 126 patients. The following manifestations were autonomic neurological abnormalities (58%) and visual disorders (50%). Nearly one-third of the cases presented cardiac abnormalities. Chinese hATTR patients were mostly early onset (AO 41.8 years), and the median time from onset to death was 7.5 years. Patients with cardiac involvement had a shorter survival duration.

## Supplementary Information

**Additional file 1: Table S1**. Quality assessment of clinical outcome studies included in the meta-analysis. **Figure S1**. Analysis of studies for mean age of onset and mean age of diagnosis of Gly83Arg mutations in transthyretin amyloidosis of different studies. The size of the squares corresponds to the weight of each study. The diamond and its width represent the pooled estimate for mean age and standard deviation (SD). **Figure S2**. Subgroup analysis for mortality by subtypes of transthyretin amyloidosis. (a) The size of the squares corresponds to the weight of each subgroup. The diamond and its width represent the pooled estimate for 2-year, all-cause mortality and 95% confidence intervals (95% CIs); (b) The size of the squares corresponds to the weight of each subgroup. The diamond and its width represent the pooled estimate for 5-year, all-cause mortality and 95% confidence intervals (95% CIs); (c) The size of the squares corresponds to the weight of each subgroup. The diamond and its width represent the pooled estimate for 10-year, all-cause mortality and 95% confidence intervals (95% CIs); hATTR-PN: hereditary transthyretin amyloid polyneuropathy; hATTR-CM and MIX: hereditary transthyretin amyloid cardiomyopathy and hereditary transthyretin amyloid cardiomyopathy and polyneuropathy.

## Data Availability

All data generated or analyzed during this study are included in this published article (and its Additional file 1).
