# Supplementary material for: Clinical phenotypes and genetic features of hereditary transthyretin amyloidosis patients in China
Source: Orphanet J Rare Dis. 2022 Sep 2;17:337. doi: 10.1186/s13023-022-02481-9 (PMC9438301; doi:10.1186/s13023-022-02481-9)
Supplement: Supplementary file 1 — Additional file 1: Table S1. Quality assessment of clinical outcome studies included in the meta-analysis. Figure S1. Analysis of studies for mean age of onset and mean age of diagnosis of Gly83Arg mutations in transthyretin amyloidosis of different studies. The size of the squares corresponds to the weight of each study. The diamond and its width represent the pooled estimate for mean age and standard deviation (SD). Figure S2. Subgroup analysis for mortality by subtypes of transthyretin amyloidosis. (a) The size of the squares corresponds to the weight of each subgroup. The diamond and its width represent the pooled estimate for 2-year, all-cause mortality and 95% confidence intervals (95% CIs); (b) The size of the squares corresponds to the weight of each subgroup. The diamond and its width represent the pooled estimate for 5-year, all-cause mortality and 95% confidence intervals (95% CIs); (c) The size of the squares corresponds to the weight of each subgroup. The diamond and its width represent the pooled estimate for 10-year, all-cause mortality and 95% confidence intervals (95% CIs); hATTR-PN: hereditary transthyretin amyloid polyneuropathy; hATTR-CM and MIX: hereditary transthyretin amyloid cardiomyopathy and hereditary transthyretin amyloid cardiomyopathy and polyneuropathy. [file 13023_2022_2481_MOESM1_ESM.docx]

**Table S1.** Quality assessment of clinical outcome studies included in the meta-analysis.

| **NO** | **STUDY** | **Representativeness** | **Selection cohort** | **Ascertainment** | **Outcome of interest** | **Comparability of cohorts** | **Assessment of outcome** | **Follow-up duration** | **Adequacy of follow-up** | **SUM** |
| --- | --- | --- | --- | --- | --- | --- | --- | --- | --- | --- |
| 1 | Xu Min^18^ | 1 | 1 | 1 | 1 | 1 | 1 | 1 | 1 | **8** |
| 2 | Li Yanfeng^13^ | 1 | 1 | 1 | 1 | 1 | 1 | 1 | 1 | **8** |
| 3 | Xi Jianying^30^ | 1 | 0 | 1 | 1 | 1 | 1 | 0 | 0 | **5** |
| 4 | Liu Jingyao^17^ | 1 | 1 | 1 | 1 | 1 | 1 | 1 | 1 | **8** |
| 5 | Zhang Yu^35^ | 1 | 0 | 1 | 1 | 1 | 1 | 0 | 0 | **5** |
| 6 | Chen Lingyan^18^ | 1 | 1 | 1 | 1 | 1 | 1 | 0 | 1 | **7** |
| 7 | Long Da^21^ | 1 | 0 | 1 | 1 | 1 | 1 | 1 | 0 | **6** |
| 8 | Zhang Amei^12^ | 1 | 1 | 1 | 1 | 2 | 1 | 1 | 1 | **9** |
| 9 | Zou Xuan^22^ | 1 | 1 | 1 | 1 | 1 | 1 | 1 | 1 | **8** |
| 10 | Xie Bing^11^ | 1 | 1 | 1 | 1 | 1 | 1 | 1 | 1 | **8** |
| 11 | Liu Tiruo^10^ | 1 | 1 | 1 | 1 | 2 | 1 | 1 | 1 | **9** |
| 12 | Yin Jie^9^ | 1 | 1 | 1 | 1 | 1 | 1 | 1 | 0 | **7** |
| 13 | Fan Junping^23^ | 1 | 1 | 1 | 1 | 1 | 1 | 1 | 1 | **8** |
| 14 | Lv Wenjuan^15^ | 1 | 0 | 1 | 1 | 1 | 1 | 1 | 1 | **7** |
| 15 | Guan Hongzhi^19^ | 1 | 0 | 1 | 1 | 2 | 1 | 1 | 1 | **8** |
| 16 | Meng Lingchao^2^ | 1 | 0 | 1 | 1 | 1 | 1 | 1 | 1 | **7** |
| 17 | Chen Hui^16^ | 1 | 1 | 1 | 1 | 1 | 1 | 1 | 1 | **8** |
| 18 | Cheng Qiusheng^16^ | 1 | 1 | 1 | 1 | 1 | 1 | 1 | 1 | **8** |
| 19 | Hu Die^27^ | 1 | 1 | 1 | 1 | 1 | 1 | 1 | 0 | **7** |
| 20 | Liu Gonglu^32^ | 1 | 1 | 1 | 1 | 2 | 1 | 1 | 1 | **9** |
| 21 | Xu Jing^33^ | 1 | 0 | 1 | 1 | 1 | 1 | 1 | 1 | **7** |
| 22 | Yang Shuo^31^ | 1 | 1 | 1 | 1 | 1 | 1 | 1 | 1 | **8** |
| 23 | Chen Chuhong^34^ | 1 | 0 | 1 | 1 | 1 | 1 | 1 | 1 | **7** |
| 24 | Zhu Peiran^14^ | 1 | 1 | 1 | 1 | 1 | 1 | 1 | 0 | **7** |
| 25 | Chen Qian^26^ | 1 | 1 | 1 | 1 | 1 | 1 | 0 | 0 | **6** |
| 26 | Hu Boling^37^ | 1 | 1 | 1 | 1 | 1 | 1 | 0 | 0 | **6** |
| 27 | Yuan Zhenhua^29^ | 1 | 1 | 1 | 1 | 1 | 1 | 1 | 0 | **7** |
| 28 | Fan Kuan^25^ | 1 | 1 | 1 | 1 | 2 | 1 | 1 | 1 | **9** |
| 29 | Miao xin^36^ | 1 | 0 | 1 | 1 | 1 | 1 | 0 | 0 | **5** |
| 30 | Qin jin^28^ | 1 | 0 | 1 | 1 | 1 | 1 | 1 | 1 | **7** |


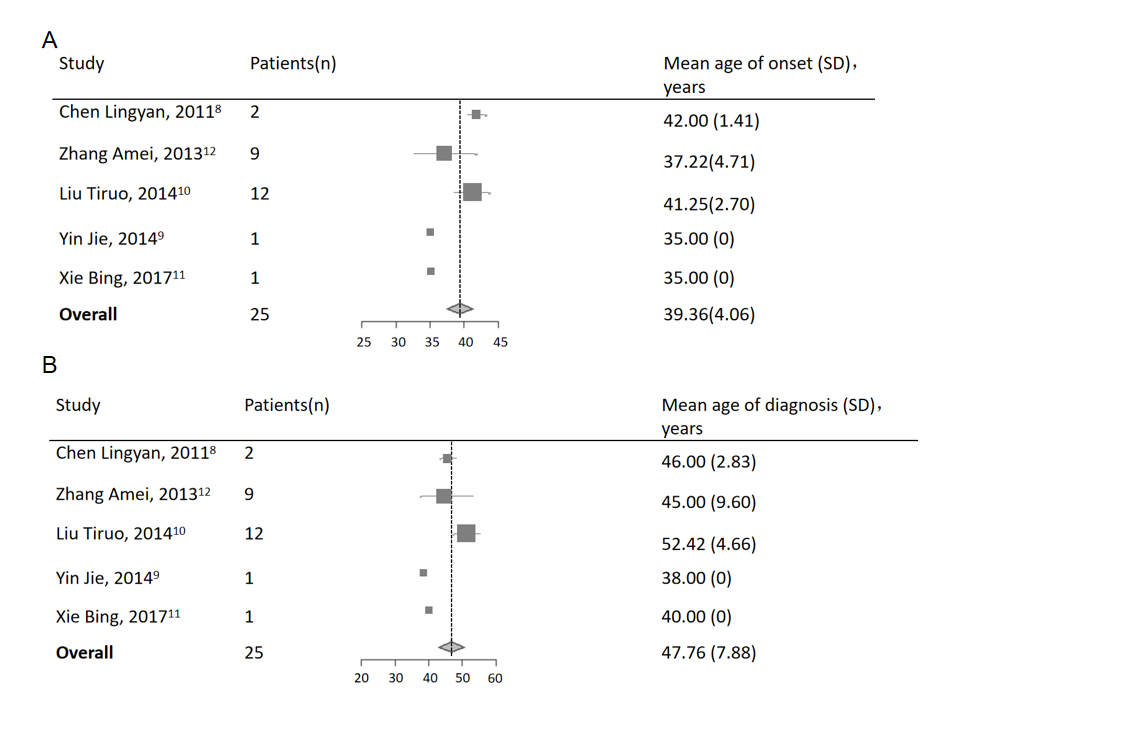


**Figure S1**. Analysis of studies for mean age of onset and mean age of diagnosis of Gly83Arg mutations in transthyretin amyloidosis of different studies. The size of the squares corresponds to the weight of each study. The diamond and its width represent the pooled estimate for mean age and standard deviation (SD).


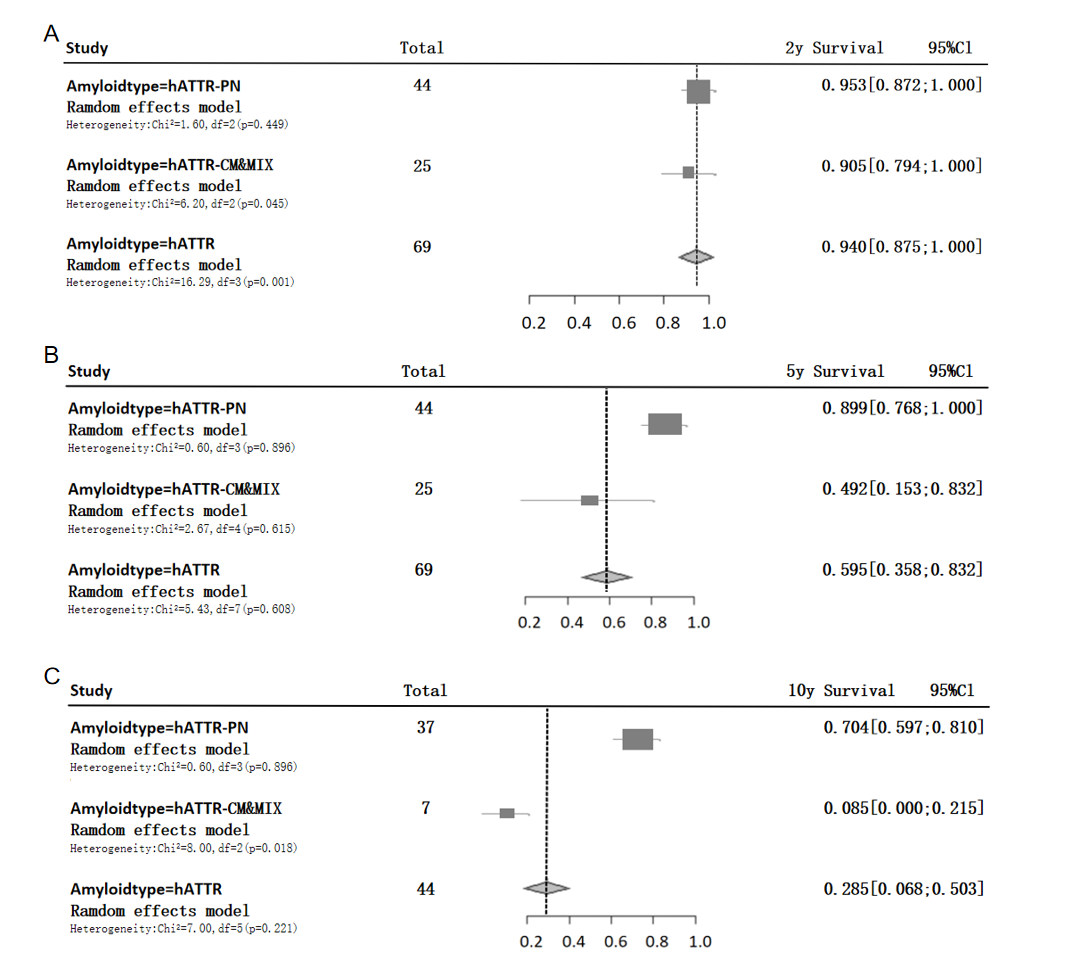


**Figure S2**. Subgroup analysis for mortality by subtypes of transthyretin amyloidosis. (a)The size of the squares corresponds to the weight of each subgroup. The diamond and its width represent the pooled estimate for 2-year, all-cause mortality and 95% confidence intervals (95% CIs); (b)The size of the squares corresponds to the weight of each subgroup. The diamond and its width represent the pooled estimate for 5-year, all-cause mortality and 95% confidence intervals (95% CIs); (c)The size of the squares corresponds to the weight of each subgroup. The diamond and its width represent the pooled estimate for 10-year, all-cause mortality and 95% confidence intervals (95% CIs);

hATTR-PN: hereditary transthyretin amyloid polyneuropathy; hATTR-CM&MIX: hereditary transthyretin amyloid cardiomyopathy & hereditary transthyretin amyloid cardiomyopathy and polyneuropathy.
